# Cannabigerol is a novel, well-tolerated appetite stimulant in pre-satiated rats

**DOI:** 10.1007/s00213-016-4397-4

**Published:** 2016-08-09

**Authors:** Daniel I Brierley, James Samuels, Marnie Duncan, Benjamin J Whalley, Claire M Williams

**Affiliations:** 1 School of Psychology and Clinical Language Sciences, University of Reading, Reading, UK; 2 School of Chemistry, Food and Pharmacy, University of Reading, Reading, UK; 3 GW Research Ltd, Cambridge, UK

**Keywords:** Appetite, Appetitive, Cachexia, Cannabigerol, Cannabis, Consummatory, Feeding, Hyperphagia, Phytocannabinoid, Tolerability

## Abstract

**Rationale:**

The appetite-stimulating properties of cannabis are well documented and have been predominantly attributed to the hyperphagic activity of the psychoactive phytocannabinoid, ∆^9^-tetrahydrocannabinol (∆^9^-THC). However, we have previously shown that a cannabis extract devoid of ∆^9^-THC still stimulates appetite, indicating that other phytocannabinoids also elicit hyperphagia. One possible candidate is the non-psychoactive phytocannabinoid cannabigerol (CBG), which has affinity for several molecular targets with known involvement in the regulation of feeding behaviour.

**Objectives:**

The objective of the study was to assess the effects of CBG on food intake and feeding pattern microstructure.

**Methods:**

Male Lister hooded rats were administered CBG (30–120 mg/kg, per ora (p.o.)) or placebo and assessed in open field, static beam and grip strength tests to determine a neuromotor tolerability profile for this cannabinoid. Subsequently, CBG (at 30–240 mg/kg, p.o.) or placebo was administered to a further group of pre-satiated rats, and hourly intake and meal pattern data were recorded over 2 h.

**Results:**

CBG produced no adverse effects on any parameter in the neuromotor tolerability test battery. In the feeding assay, 120–240 mg/kg CBG more than doubled total food intake and increased the number of meals consumed, and at 240 mg/kg reduced latency to feed. However, the sizes or durations of individual meals were not significantly increased.

**Conclusions:**

Here, we demonstrate for the first time that CBG elicits hyperphagia, by reducing latency to feed and increasing meal frequency, without producing negative neuromotor side effects. Investigation of the therapeutic potential of CBG for conditions such as cachexia and other disorders of eating and body weight regulation is thus warranted.

## Introduction

*Cannabis sativa* L. has been utilised for medicinal and recreational purposes for millennia and is increasingly being recognised as a valuable source of unique compounds (phytocannabinoids) with a multitude of potential therapeutic applications (Deiana et al. 2012). The main psychoactive constituent of *C. sativa*, ∆^9^-tetrahydrocannabinol (∆^9^-THC), was first isolated and characterised in the 1960s (Mechoulam & Gaoni 1965; Gaoni & Mechoulam 1971), and this has been followed by the discovery of numerous additional phytocannabinoids (pCBs) over the last two decades (see Mechoulam & Hanus 2000 for review). Over 100 pCBs have now been isolated from *C. sativa* (Elsohly & Slade 2005), but despite their structural similarities, the pCBs show considerable heterogeneity in their pharmacological targets and/or physiological activities (Hill et al. 2012a).

One of the better known properties of *C. sativa* is its ability to stimulate appetite (hyperphagia), which has been described anecdotally by recreational users and demonstrated under laboratory conditions (Hollister 1971; Mattes et al. 1994). In rodent models, our laboratory has shown that oral administration of ∆^9^-THC to pre-satiated rats produced significant short-term hyperphagia (Williams et al. 1998), characterised by a marked reduction in latency to begin feeding (Williams and Kirkham 2002a), an effect that was reversed by co-administration of the selective CB_1_ receptor (CB_1_R) antagonist SR141716 (Williams and Kirkham 2002b). Similarly, CB_1_R-mediated hyperphagic effects have also been observed following administration of the endocannabinoids (eCB) anandamide (AEA) (Williams & Kirkham 1999) and 2-arachidonoylglyerol (2-AG) (Kirkham et al. 2002). Importantly, in the latter study, we also demonstrated altered brain levels of AEA and 2-AG during fasting and feeding states, implicating eCBs in the control of appetite.

In the years since these findings, the role of the eCB system in appetite regulation and energy balance has been the subject of intensive research and is now emerging as a major target area for a variety of metabolic disorders (see Di Marzo et al. 2011 for review). Despite promising therapeutic potential, treatments directly targeting CB_1_R-mediated appetite regulation have, thus far, had limited clinical success. The CB_1_R antagonist SR141716 was licenced as an anti-obesity treatment in Europe in 2006; however, it was withdrawn 2 years later due to adverse side effects including depression and suicidality (Derosa and Maffioli 2012). Synthetic ∆^9^-THC (dronabinol) is licenced for treatment of chemotherapy-induced nausea and vomiting; however, it is not recommended as a first-line treatment due to adverse side effects associated with its psychoactivity (Todaro 2012). Two clinical trials conducted on ∆^9^-THC for appetite stimulation in cancer cachexia patients did not show positive results, possibly due to the low doses required to attain an acceptable tolerability profile (Jatoi et al. 2002; Strasser et al. 2006).

Recently, we have started investigating the effects of the non-∆^9^-THC pCBs on feeding behaviour (Farrimond et al. 2010a; Farrimond et al. 2010b; Farrimond et al. 2012a; Farrimond et al. 2012b; Brierley et al. 2016). This work suggests that some pCBs may offer potential for therapeutic appetite regulation without the psychoactive side effect profile of ∆^9^-THC-containing preparations. In the present study, we investigated the effects of one such pCB, cannabigerol (CBG), which does not produce ‘cannabimimetic’ psychoactive side effects and is hence typically described as non-psychoactive (Mechoulam et al. 1970). CBG is a relatively little-studied biosynthetic precursor (in *C. sativa*) of the major pCBs ∆^9^-THC and cannabidiol (CBD) (Hill et al. 2012a). Pharmacodynamic studies of CBG in vitro have determined that it acts as a potent α2-adrenoceptor agonist (EC_50_ = 0.2 nM) and a modest 5-HT_1A_R competitive antagonist (at 10 μM) and can weakly bind, but not activate, CB_1_R and CB_2_R (*K*_*i*_ = 81 and 2600 nM, respectively) (Cascio et al. 2010; Pertwee et al. 2010). Furthermore, it has been shown to inhibit the reuptake of the endocannabinoid AEA (IC_50_ = 11.3 μM) and interacts with a number of transient receptor potential (TRP) channels, acting as an agonist at TRPA1, TRPV1 and TRPV2 (EC_50_ = 0.7, 1.3 and 1.7 μM, respectively) and as a potent antagonist at TRPM8 (IC_50_ = 0.16 μM) (De Petrocellis et al. 2011). CBG has also recently been shown to block voltage-gated sodium channels Na_v_ 1.1, 1.2 and 1.5 (IC_50_ = 88, 79 and 36 μM, respectively) (Hill et al. 2014). Given that CBG readily penetrates the blood brain barrier (Deiana et al. 2012) and interacts with a number of eCB and non-eCB targets with known involvement in the control of feeding and energy balance (Halford et al. 2004; Di Marzo and Matias 2005; Lee et al. 2015), the in vitro data available suggest this that pCB could conceivably elicit a centrally mediated stimulation of feeding behaviour. Although there is a paucity of in vivo studies of CBG in general, and feeding studies in particular, it has been reported that an acute low dose (2.5 mg/kg, i.p.) administered to rats enhanced saccharin palatability in a taste reactivity test (O’Brien et al. 2013). As part of one of our previous studies, we investigated the effects on feeding behaviour of low doses of CBG (0.176–17.6 mg/kg, p.o.), which were scaled to the concentrations found in a low-∆9-THC cannabis extract which induced hyperphagia (Farrimond et al. 2012b). In that study however, despite a suggestion of increased food intake over 2 h, no significant effects of CBG on appetite were found.

The present study was thus conducted with the aim of investigating whether CBG was able to stimulate appetite, at higher doses than previously tested. To ensure that any potential therapeutic utility of CBG would not be compromised by detrimental neuromotor side effects, our first experiment comprised a neuromotor tolerability test battery to assess the pCB’s effects on locomotor activity, balance, fine motor control and muscular strength. As CBG has previously been found to have no cannabimimetic effects in the mouse tetrad assay up to a maximal tested dose of 80 mg/kg (El-Alfy et al. 2010), had minimal behavioural effects (at 3–100 mg/kg) in a mouse Irwin assay (Duncan et al. 2014) and no signs of acute toxicity were reported in a pharmacokinetic study of 120-mg/kg doses (Deiana et al. 2012), a dose range of 30–120 mg/kg was used in this tolerability experiment. Subsequently, a second experiment was conducted using a pre-feed satiation paradigm to assess the acute hyperphagic effects of CBG. Results from a pilot of this experiment suggested that CBG may elicit dose-dependent hyperphagia, which did not appear to have a ceiling effect up to 120 mg/kg, and so an additional 240-mg/kg dose group was included in the design of the experiment. Infrared activity monitoring was performed concurrently throughout the feeding experiment to corroborate the effects of CBG on locomotor activity determined during the tolerability battery and to extend this investigation over a longer time frame and higher dose range.

## Methods

### Experiment 1: effects of CBG in a neuromotor tolerability test battery

#### Drugs

CBG (GW Pharmaceuticals, UK) was dissolved directly into sesame seed oil (by magnetic stirring at 57 °C) to produce a maximal working concentration of 120 mg/ml. Working solutions of 60 and 30 mg/ml were then prepared by serial dilution. CBG solutions were prepared freshly on each test day and protected from light until administration.

Doses of CBG or sesame seed oil vehicle alone were administered using a within-subject design, with all experimental units (individual animals) receiving 0, 30, 60 and 120 mg/kg CBG according to a pseudo-random, counterbalanced, Latin square protocol. All animals received doses separated by a minimum 48-h washout period. On test days, animals were administered CBG or vehicle 60 min prior to commencement of testing. CBG or sesame seed oil vehicle was administered per ora (p.o.) via a syringe placed into the cheek pouch at 1-ml/kg dosing volume.

#### Animals

Twelve young adult male Lister Hooded rats (Harlan, UK), weighing 200–225 g on delivery, were housed in pairs in temperature and humidity-controlled rooms with reversed light cycles (dim red light 12:00–24:00), with standard laboratory chow and water available ad libitum.

#### Procedure

Prior to testing, animals were subjected to a 5-day habituation process, consisting of daily handling, vehicle drug administration, habituation to open field and static beam test procedures. On test days, all procedures were conducted during the first half of the dark period (12:00–18:00) in the same room as the animals were housed. All test equipment was cleaned with 70 % ethanol and allowed to dry completely between animals. All tasks were presented in the order below with animals having a 5-min rest period in their home cage between tasks.

#### Open field

Consisting of a 1.1 × 1.1 × 0.4-m black acrylic-lined box, delineated into a 5 × 5 square grid and comprising a 3 × 3 central sector and a single square-wide peripheral sector, the open field was illuminated by dim red light (∼10 lx). Animals were placed in a consistent corner of the open field, and behaviour was video recorded for 5 min. Videos were analysed offline using Observer XT software (Noldus, Netherlands). Locomotor activity was quantified based on the number of times animals crossed the lines on the open field floor, with time spent in the central area of the field and latency to first entry used to quantify anxiety-like behaviour (i.e. degree of thigmotaxis). It should be noted that the habituation period animals received to the open field component of the test battery is necessary for within-subject assessment of drug-induced changes of locomotor activity. However, as a consequence, the aversive/novel nature of the environment is attenuated in comparison to the non-habituated version of this task, which is primarily used to assess anxiety-like behaviour.

#### Static beam

The apparatus consisted of a 3.2-cm-diameter cylindrical beam, 1 m in length and suspended 0.5 m above floor level. A bright light was positioned at the start of the beam and an enclosed goal box at the end. Animals were placed at the start of the beam and allowed a maximum of 5 min to successfully traverse its length to reach the goal box. Animals were then given a 2-min rest period in home cages prior to repeating the test. Tests were video recorded for off-line coding using Observer XT software (Noldus, Netherlands). In the static beam test, performance generated four outcome measures, based on successful completion or length of beam traversed prior to falling (pass rate and distance travelled), number of times paws were fully extended past the beam (foot slips) and time taken to traverse the middle 50 cm of beam (speed).

#### Forelimb grip strength

Animals completed two repeats of the forelimb grip strength test, separated by a 30-s rest period. Animals were placed with forelimbs gripping a trapeze bar connected to a digital force gauge (FH50, Sauter GmbH, Germany), then uniformly pulled by the tail base away from bar along the horizontal plane until grip was released and peak force recorded.

#### Forelimb grip strength

Analysis All behavioural coding was conducted by an experimenter blinded to treatment allocation. For static beam and forelimb grip strength outcome measures, where animals were subjected to two tests during the battery, data represent the mean of the two technical repeats, with the exception of pass rate on static beam in which a score of 0–2 was allocated based on number of successfully completed tests. All continuous data were analysed using SPSS 18 (IBM, UK) by one-way repeated measures ANOVA (ordinal pass rate data were analysed by Friedman’s ANOVA), with degrees of freedom and *p* values corrected, where assumptions of sphericity were violated (using Greenhouse-Geisser correction). When significant overall dose effects were observed, planned comparisons of all dose groups vs vehicle group were conducted to reveal any significant pairwise comparisons. Results were considered significant if *p* < 0.05.

### Experiment 2: effects of CBG on feeding behaviour

#### Drugs

Briefly, on each test day, CBG (GW Pharmaceuticals, UK) was dissolved in sesame seed oil and then serially diluted to produce working solutions of 240, 120, 60 and 30 mg/ml. Using a within-subject, counterbalanced, repeated measure design, doses of CBG or vehicle were orally administered to animals as described in experiment 1. Each test day was separated by a minimum 48-h washout.

#### Animals

Sixteen young adult male Lister Hooded rats (Harlan, UK), weighing 200–225 g on delivery, were housed in pairs in temperature and humidity-controlled rooms with reversed light cycles (dim red light 12:00–24:00), with standard laboratory chow and water available ad libitum.

#### Procedure

Acute feeding experiments were conducted in pre-satiated animals using our well-established paradigm for the detection of hyperphagia following administration of cannabinoids (Williams et al. 1998). Additionally, concurrent measurement of ambulatory activity and rearing during the feeding test protocol was conducted, using two levels of infrared photobeam activity sensors arrayed around the test cages.

Prior to the start of testing, animals were habituated to handling (10 days), vehicle dosing and the pre-feed procedure (7 days) and the testing apparatus (5 days). The pre-feed procedure was conducted at the onset of the dark period, when animals were transferred to individual cages containing 30.5 ± 0.5 g of highly palatable wet-mash food. The wet-mash comprised 1-part rat and mouse expanded ground diet (SDS, Witham, UK) and 1.25-part tap water. Animals were allowed 2 h to consume the wet-mash, following which they were returned to their home cages and quantity of wet-mash consumed was measured. Animals were habituated to this pre-feed procedure until a stable consumption level was reached, as indicated by a non-significant main effect of test day by one-way ANOVA across four consecutive habituation days (*F*_3, 63_ = 0.5603, *p* = 0.644), with mean consumption during these days being 19.9–20.5 g.

On test days, immediately after the pre-feed procedure, animals were administered doses of CBG or vehicle and returned to their home cages for 1 h to allow for drug assimilation, during which time food was unavailable. Animals were then placed into feeder cages for 2 h, during which time food consumption and locomotor activity were recorded on automated food intake (TSE Systems, Germany) and infrared photobeam activity systems (Ugo Basile, Italy) and behaviour was video recorded. At the end of the experiment, animals were returned to their home cages, with food available ad libitum until the following test procedure ≥48 h later.

Quantity of food consumed during the 2 h test was confirmed manually by weighing the remaining chow pellets in the food hoppers and any crumbs in spillage trays below the cages and subtracting these from the initial weight of chow in the hopper. The automated food intake system provided data output on the time, duration and size of each feeding bout, which were confirmed from video recordings as genuine feeding episodes as opposed to exploratory interactions with food hoppers. Feeding bouts were combined into ‘meals’, defined as feeding bouts consuming ≥0.5 g and separated by ≥900 s, criteria previously shown to more accurately reflect the natural process of food consumption (Williams & Kirkham 2002a; Farrimond et al. 2012b).

#### Analysis

Data were analysed to provide measures of feeding behaviours during appetitive and consummatory phases, using the parameters of latency to first meal and meal frequency (appetitive) and meal sizes and durations (consummatory) in addition to hourly and total intake quantities. Ambulatory locomotor activity was quantified over the test duration using the number of infrared beam breaks. All continuous data were analysed using SPSS 18 (IBM, UK) by one-way repeated measures ANOVA, with degrees of freedom and *p* values corrected where assumptions of sphericity were violated (using Greenhouse-Geisser correction). When significant overall dose effects were observed, planned comparisons of all dose groups vs vehicle group were conducted to reveal any significant pairwise comparisons. Results were considered significant if *p* < 0.05.

All experiments were performed at the University of Reading in accordance with the principles of laboratory animal care, UK Home Office regulations [Animals (Scientific Procedures) Act 1986] and the ARRIVE guidelines for reporting experiments involving animals (Kilkenny et al. 2010; McGrath et al. 2010).

## Results

### Experiment 1: effect of CBG in a neuromotor tolerability test battery

#### Open field test

General ambulatory activity in the open field test was not modulated by administration of CBG at any dose (Table 1), as determined by the number of line crosses observed (*F*_3, 27_ = 0.454, *p* = 0.716). Similarly, the lack of significant dose effect on either duration spent in the central sector (*F*_1.9, 17.6_ = 1.80, *p* = 0.195) or the latency to enter the central sector (*F*_3, 27_ = 0.262, *p* = 0.852) suggests that CBG does not have any effect on anxiety-like behaviour in this version of the test.

**Table 1:** Behavioural parameters in the habituated open field and forelimb grip strength test components of the neuromotor tolerability test battery (Experiment 1). Administration of CBG at doses up to 120 mg/kg had no deleterious effects on locomotor activity or grip strength performance nor any effect on anxiety-like behaviours. Data presented as mean ± SEM and analysed by one-way repeated measures ANOVA, all groups *n* = 12

	CBG (mg/kg)			
	0	30	60	120
Open field test				
Line crosses	136 (±18)	145 (±14)	142 (±6)	130 (±10)
Central sector duration (s)	23.1 (±4.7)	26.0 (±3.9)	33.1 (±5.9)	19.2 (±2.7)
Latency to central sector entry (s)	74.7 (±21.4)	70.2 (±24.3)	64.9 (±17.7)	85.9 (±20.7)
Grip strength test				
Grip strength (kgf)	0.811 (±0.062)	0.747 (±0.044)	0.762 (±0.032)	0.740 (±0.051)

#### Static beam test

**Fig. 1:**
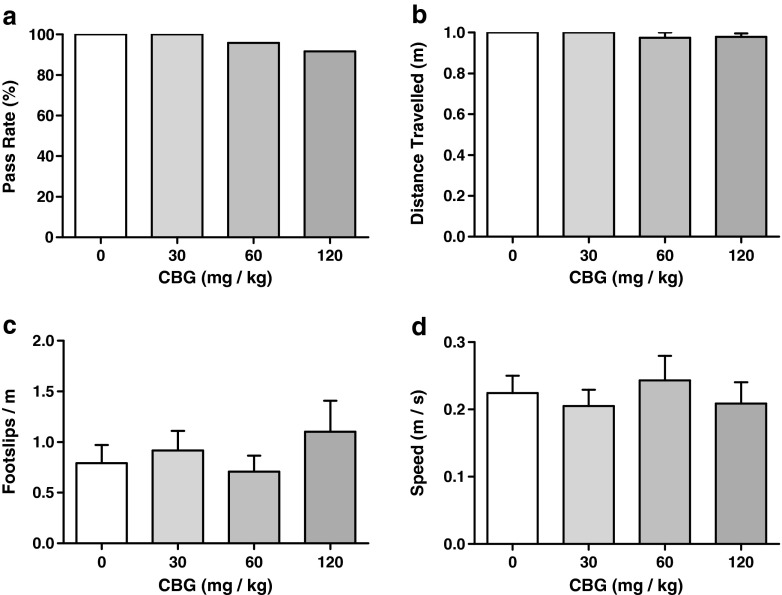
Performance parameters in the static beam test component of the neuromotor tolerability test battery (Experiment 1). Administration of CBG at doses up to 120 mg/kg had no deleterious effects on measures of balance (**a**, **b**) or fine motor control (**c**, **d**). Data presented as mean ± SEM and analysed by one-way repeated measures ANOVA, all groups *n* = 12

**Fig. 2:**
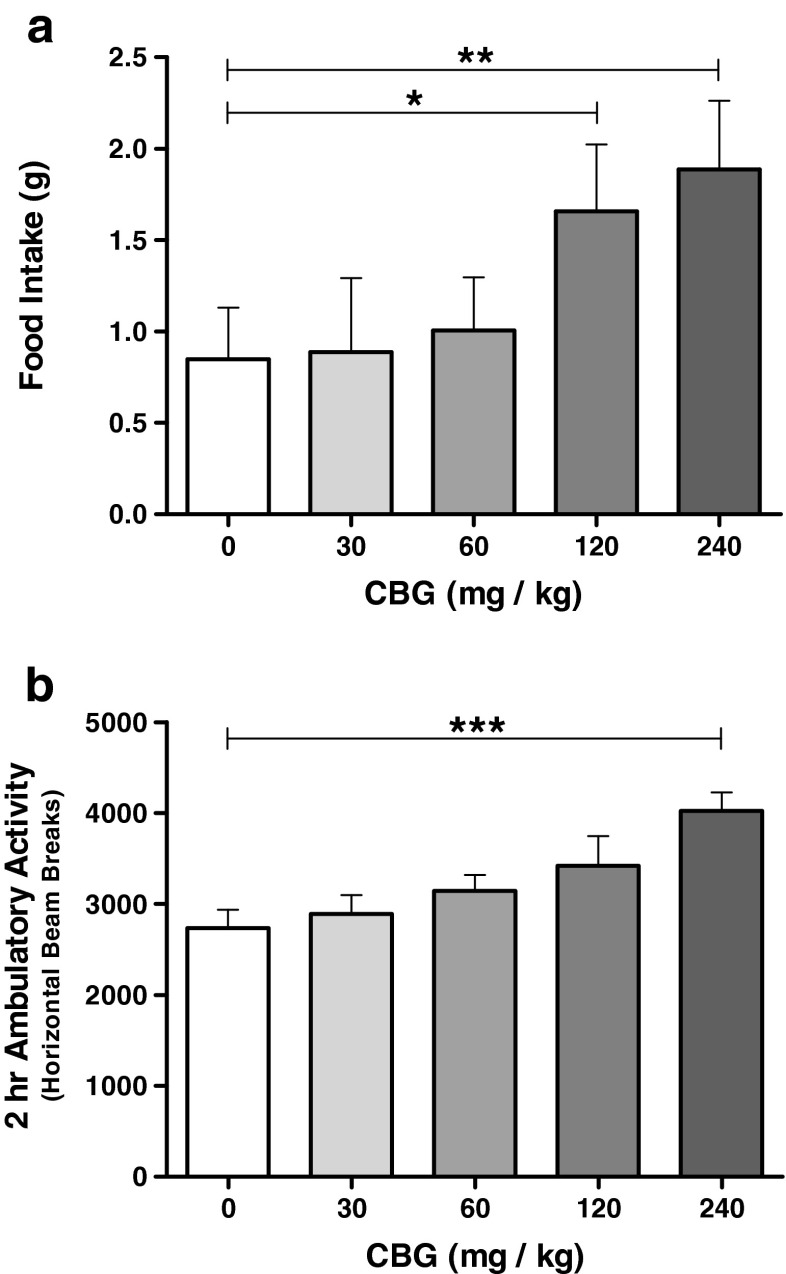
Total food intake and locomotor activity levels during the feeding behaviour test (Experiment 2). Administration of CBG at 120 and 240 mg/kg increased food intake (**a**) and at 240 mg/kg increased locomotor activity (**b**). Data presented as mean ± SEM and analysed by one-way repeated measures ANOVA and planned comparisons. All groups *n* = 16. *p < 0.05, **p < 0.01, ***p < 0.001

CBG had no effect on any measure of balance or motor coordination as assessed in the static beam test. Gross measures of balance (Fig. 1a, b) were unaffected, as demonstrated by non-significant effects of dose on pass rate (*F*_*r*3_ = 3.667, *p* = 0.30) and distance travelled (*F*_1.5, 16.9_ = 0.758, *p* = 0.451). Measures of fine motor coordination (Fig. 2c, d) were similarly unaffected, with non-significant effects of dose observed on the number of foot slips (*F*_1.5, 16.6_ = 0.687, *p* = 0.477) and speed across the beam (*F*_3,33_ = 0.699, *p* = 0.560).

#### Grip strength test

In the forelimb grip strength test for muscular strength and functional neurotoxicity (Table 1), CBG also had no significant effect on performance at any dose level (*F*_3, 33_ = 0.564, *p* = 0.643).

These data from the neuromotor tolerability test battery extend the previous limited data in the literature to show that acute oral doses of CBG up to 120 mg/kg do not elicit any detrimental motoric side effects. On the basis of these findings, we decided to conduct the feeding behaviour study (Experiment 2) using the full dose range in Experiment 1 and an additional higher-dose group (240 mg/kg), with 2-h ambulatory activity measured concurrently to corroborate the open field data and assess if any sedative/motoric effect was apparent at the highest dose level and/or over a longer test duration.

### Experiment 2: effect of CBG on feeding behaviour

#### Hourly food intake

The effectiveness of the pre-feed procedure was evident by the very low baseline intake level in the vehicle group, which maximises the opportunity to detect drug-induced hyperphagia. The total quantity of food consumed during the test period was increased following CBG administration (Fig. 2a) in a dose-dependent manner (*F*_4, 60_ = 3.967, *p* = 0.006). Overall, animals consumed 1.66 (±0.37) g following 120 mg/kg and 1.89 (±0.38) g following 240 mg/kg CBG (*F*_1, 15_ = 5.328, *p* = 0.036 and *F*_1, 15_ = 8.909, *p* = 0.009, respectively) compared to 0.85 (±0.28) g for vehicle-treated animals. When broken down by hourly consumption, a significant effect of CBG was observed for hour 1 intake (*F*_4, 60_ = 2.607, *p* = 0.044); however, no post hoc comparisons were significant, with only the 120-mg/kg group nearing significance (*F*_1, 15_ = 3.741, *p* = 0.072). In hour 2, a significant effect of CBG was observed (*F*_4, 60_ = 2.722, *p* = 0.038), with vehicle-treated animals consuming 0.38 (±0.18) g, compared to 0.99 (±0.19) g following 240 mg/kg CBG (*F*_1, 15_ = 11.538, *p* = 0.004).

#### Analysis of meal microstucture

**Fig. 3:**
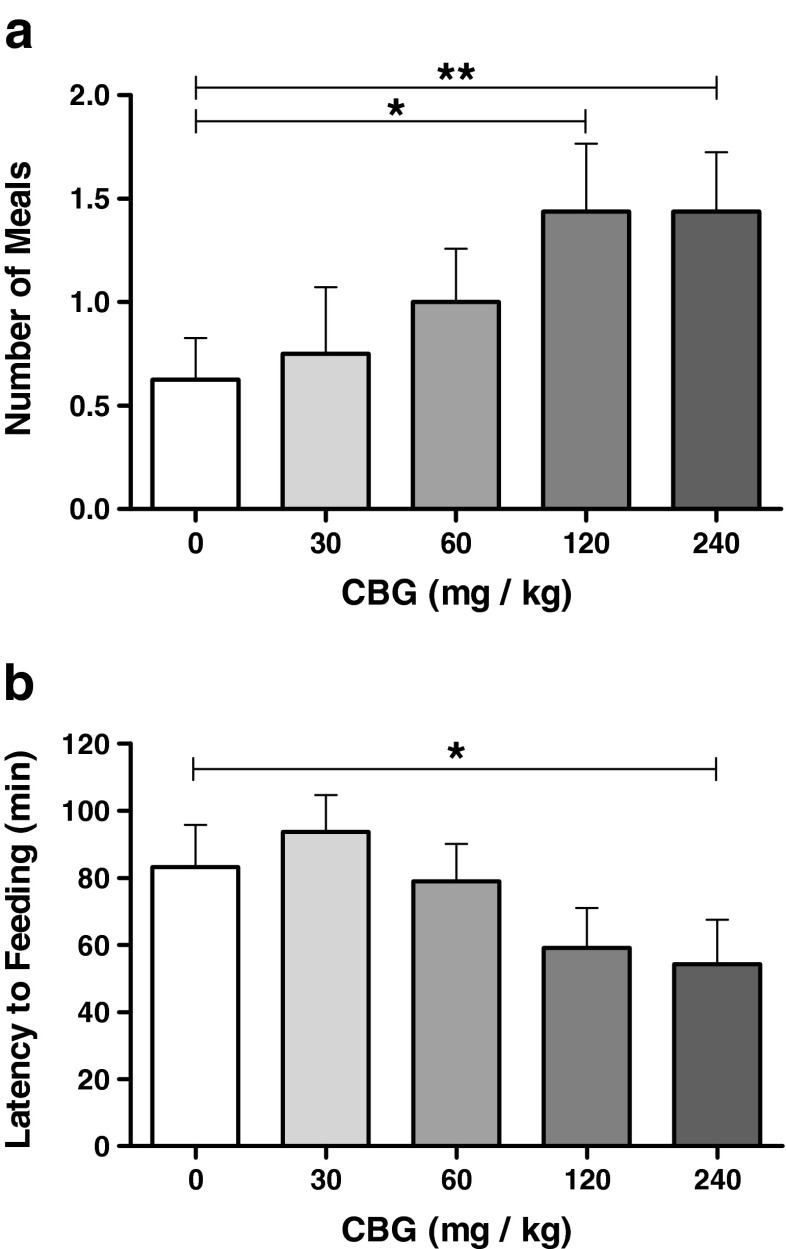
Appetitive phase feeding behaviour parameters in the feeding behaviour test (Experiment 2). Administration of CBG at 120 and 240 mg/kg increased the number of meals consumed (**a**) and at 240 mg/kg reduced the latency to onset of feeding (**b**). Data presented as mean ± SEM and analysed by one-way repeated measures ANOVA and planned comparisons. All groups *n* = 16. *p < 0.05, **p < 0.01

A more granular analysis of meal microstructure following CBG administration revealed significant stimulatory effects on feeding frequency and latency to feed (consistent with appetitive stimulation), however only modest effects on intra-meal factors consistent with consummatory stimulation (Fig. 3 and Table 2). CBG treatment produced a significant increase in the number of meals consumed during the test (*F*_4, 60_ = 3.306, *p* = 0.016; Fig. 3a). On average, our pre-feed procedure was so successful that vehicle-treated animals consumed less than 1 meal (0.63 ± 0.20) during the test with only 7/16 animals consuming any food at all and no animal consuming more than 2 meals. In comparison, animals treated with 120 and 240 mg/kg CBG consumed more than twice that average number of meals (1.44 ± 0.33 [*F*_1, 15_ = 7.752, *p* = 0.014] and 1.44 ± 0.29 [*F*_1, 15_ = 12.739, *p* = 0.003], respectively), with 12/16 animals consuming at least 1 meal and some consuming up to 4.

**Table 2:** Hourly food intake and meal pattern microstructure parameters in the feeding behaviour test (Experiment 2)

	CBG (mg/kg)				
	0	30	60	120	240
Hourly food intake (g)					
Hour 1	0.47 (±0.22)	0.40 (±0.25)	0.55 (±0.25)	1.06 (±0.30)	0.89 (±0.25)
Hour 2	0.38 (±0.18)	0.49 (±0.20)	0.46 (±0.17)	0.59 (±0.15)	0.99** (±0.19)
Total	0.85 (±0.28)	0.89 (±0.40)	1.01 (±0.29)	1.66* (±0.37)	1.89** (±0.38)
Meal size (g)					
Meal 1	0.65 (±0.23)	0.38 (±0.16)	0.57 (±0.19)	0.93 (±0.18)	1.04 (±0.23)
Meal 2	0.20 (±0.11)	0.30 (±0.15)	0.22 (±0.09)	0.57 (±0.23)	0.64 (±0.18)
Meal 1 + 2	0.85 (±0.28)	0.68 (±0.30)	0.79 (±0.24)	1.51 (±0.31)	1.68* (±0.34)
Meal duration (min)					
Meal 1	5.9 (±2.7)	1.1 (±0.7)	3.1 (±1.2)	4.0 (±1.1)	5.9 (±1.9)
Meal 2	0.3 (±0.2)	0.8 (±0.5)	0.5 (±0.3)	2.4 (±1.5)	2.9 (±1.1)
Meal 1 + 2	6.2 (±2.7)	1.9 (±1.1)	3.6 (±1.3)	6.4 (±1.8)	8.7 (±2.3)
All meals	6.2 (±2.7)	3.0 (±1.5)	3.6 (±1.3)	8.7 (±2.7)	9.1 (±2.3)

Following administration of 240 mg/kg CBG, hour 2 and total food intake were increased, as was the size of meal 1 + 2. Total consumption was also increased following administration of 120 mg/kg CBG. Data presented as mean ± SEM and analysed by one-way repeated measures ANOVA and planned comparisons. All groups *n* = 16

*p < 0.05

**p < 0.01

Given that most animals consumed two meals or fewer, particularly in vehicle and low-dose CBG groups, we decided to further investigate feeding behaviours during the consummatory phase by analysing the size and duration of the first two meals consumed, both individually and cumulatively (Table 2). A significant effect of CBG on the size of meal 1 was observed (*F*_4, 60_ = 2.630, *p* = 0.043); however, no significant comparisons were revealed. No significant effect of CBG was observed on the size of meal 2 (*F*_4, 60_ = 2.124, *p* = 0.089); however, a significant effect of CBG was observed on the cumulative size of these two meals (*F*_4, 60_ = 3.927, *p* = 0.007). Whilst baseline intake in meals 1 + 2 was 0.85 (±0.28) g, animals administered 120 mg/kg CBG consumed 1.51 (±0.31) g (*F*_1, 15_ = 4.490, *p* = 0.051) and those administered 240 mg/kg CBG consumed 1.68 (±0.34) g (*F*_1, 15_ = 6.951, *p* = 0.019) during these two meals. In contrast, once feeding had started, the duration of feeding was not significantly affected by CBG administration (see Table 2), with no significant effect of CBG evident on the duration of meal 1 (*F*_2.1, 31.6_ = 1.628, *p* = 0.211) or meal 2 (*F*_2.0, 30.0_ = 1.827, *p* = 0.178). A significant dose effect was observed on the cumulative duration of these meals (*F*_4, 60_ = 2.626, *p* = 0.043); however, no significant comparisons were revealed. No significant effect of dose was observed on the total duration of feeding (*F*_2.4, 37.1_ = 2.931, *p* = 0.055). To investigate the appetitive aspect of feeding behaviour, we analysed the latency to the onset of feeding (Fig. 3b), which was significantly modulated by CBG (*F*_4, 60_ = 3.124, *p* = 0.021). Administration of 240 mg/kg CBG reduced the latency to feeding by approximately 30 min compared with vehicle-treated animals (*F*_1,15_ = 7.285, *p* = 0.016), for which the mean feeding onset was at ∼80 min. Whilst similar patterns were seen with the 120-mg/kg dose, no significant effect was seen (*F*_1,15_ = 3.651, *p* = 0.075).

Overall, these data from experiment 2 demonstrate that administration of CBG at 120–240 mg/kg elicits hyperphagia even under conditions designed to minimise food intake. This dose-dependent hyperphagia was primarily driven by stimulation of behaviours during the appetitive phase, causing animals to begin feeding sooner and eat more meals, resulting in greater overall food intake during the test period.

#### Hourly locomotor activity

To corroborate and extend the investigation of the effects of CBG on general locomotor activity in Experiment 1, we concurrently measured ambulatory and rearing behaviour in the feeding test cages throughout the duration of Experiment 2 to establish the effects of CBG over an extended time period and greater dose range. Whilst the absence of any detrimental sedative effect of CBG (up to 120 mg/kg) from the open field test was confirmed, interestingly, CBG in fact produced a significant stimulant effect on locomotor activity over the 2-h duration of the feeding test (Fig. 2b). The significant dose effect of CBG (*F*_4, 60_ = 7.121, *p* < 0.0005) was due to an almost 50 % increase in ambulatory activity in animals administered 240 mg/kg CBG (*F*_1, 15_ = 58.325, *p* < 0.0005), although activity in animals administered 30–120-mg/kg doses was not significantly increased. CBG administration did not have any effect on exploratory rearing behaviour over the 2-h test duration (*F*_2.3, 33.8_ = 2.853, *p* = 0.066).

## Discussion

The data presented here demonstrate a novel activity of CBG, as an appetite stimulant at 120–240 mg/kg. At these doses, CBG increased food intake, predominantly via stimulation of appetitive phase feeding behaviours. Furthermore, CBG acutely administered at doses ≤120 mg/kg did not elicit any detrimental neuromotor effects on locomotor activity, balance, fine motor control or muscular strength, and at 240 mg/kg appears to have some stimulant activity.

The widely utilised and validated feeding paradigm employed in this study is designed to sensitively detect even relatively small hyperphagic actions of cannabinoid agents (Williams et al. 1998). The paradigm provides detailed analysis of meal microstructure, allowing quantification of discrete behaviours during the appetitive and consummatory phases of feeding. The incorporation of an infrared locomotor activity monitoring system provides additional measures of ambulatory activity and rearing behaviour, allowing differentiation of locomotion-dependent and locomotion-independent effects of drugs on feeding behaviour. The use of this paradigm in the present study allows the direct comparison of the effects of CBG to previously published results using the same paradigm for the eCB anandamide (Williams and Kirkham 2002a); the purified pCBs ∆^9^-THC, CBD, cannabiniol (CBN) and cannabidiolic acid (Williams et al. 1998; Williams and Kirkham 2002b; Williams and Kirkham 2002a; Farrimond et al. 2012b; Brierley et al. 2016); and low- and non-∆^9^-THC cannabis extracts and analogues (Farrimond et al. 2010a; Farrimond et al. 2010b; Farrimond et al. 2012a). It should be noted that, unlike these previous studies of ∆^9^-THC, the doses of CBG used in the present study are considerably higher than concentrations typically found in whole *C. sativa* preparations. As such, the hyperphagic activity of CBG reported here is unlikely to meaningfully contribute to the appetite-stimulating effects of cannabis consumption in humans.

In this study, administration of CBG at 120–240 mg/kg dose dependently increased total food intake over the 2-h test period. This is in contrast to previous studies of various ∆^9^-THC formulations, which elicit a robust increase in intake during hour 1 followed by a compensatory decrease during hour 2 (Farrimond et al. 2010a; Farrimond et al. 2012a). The pCB CBN elicits a similar biphasic effect on food intake during this 2-h paradigm, with hyperphagia blocked by the CB_1_R antagonist SR141716, indicative of a ∆^9^-THC-like mechanism of action (Farrimond et al. 2012b). In our study, CBG also increased appetitive phase feeding behaviour, with the onset of feeding advanced by approximately 30 min, from 83 to 54 min. However, this is somewhat in contrast to previous studies of ∆^9^-THC formulations and CBN, in which feeding was initiated within 10–20 min, despite similar long latencies in vehicle groups (Farrimond et al. 2010a; Farrimond et al. 2012a; Farrimond et al. 2012b). Hence, it appears that whilst CBG may stimulate the appetitive component of feeding behaviour, it does so to a lesser degree than ∆^9^-THC and CBN.

Whilst the CBG-induced increase in feeding frequency and decrease in latency are consistent with stimulation of the appetitive component of feeding, the modest effects on intra-meal factors provide little evidence for stimulation of the consummatory component. Given that a significant effect of CBG was only evident on the cumulative size of meals 1 and 2, it is apparent that increased consumption is predominantly driven by the dose-dependent increase in feeding frequency, rather than significant increase in individual meal sizes. Similarly, the lack of significantly increased durations of individual meals does not support a stimulatory effect of CBG on the consummatory component of feeding behaviour. Differences are thus again evident between consummatory meal microstructure parameters following administration of CBG, and those of ∆^9^-THC formulations, which are typified by robust increases in both the size and duration of the first meal consumed (Farrimond et al. 2010a). Considered overall, the alterations in food intake and meal pattern microstructure induced by CBG demonstrate a dose-dependent hyperphagic effect, predominantly mediated by stimulation of the appetitive component of feeding behaviour.

Such differences in patterns of feeding behaviour stimulation between CBG and pCBs acting directly as CB_1_R agonists are consistent with the limited in vitro pharmacodynamic data on CBG, which have shown that whilst it has some affinity for this receptor, it does not appear to activate it (Cascio et al. 2010; Pertwee et al. 2010). Given that CBG has been shown to be one of the most effective pCBs at inhibiting AEA reuptake (De Petrocellis et al. 2011), it is instead possible that it elicits CB_1_R-mediated hyperphagia in an indirect manner, via upregulation of orexigenic endocannabinoid tone (Kirkham et al. 2002; Reyes-Cabello et al. 2012). The TRPV1 agonist activity of CBG could conceivably contribute to such a mechanism, given the recent observation that TRPV1 agonists can themselves inhibit AEA reuptake (Hofmann et al. 2014). Alternatively, CBG-induced hyperphagia may be mediated by its activity (to date only observed in vitro) as a highly potent agonist of α2-adrenoceptors (Cascio et al. 2010). Consistent with this, stimulation of α2-adrenoceptors in the hypothalamic paraventricular nucleus has been shown to have hyperphagic effects in satiated rats (Wellman et al. 1993; Taksande et al. 2011), whilst administration of the α2-adrenoceptor agonist clonidine into the median raphe nucleus had orexigenic effects in free feeding (Mansur et al. 2010) but not fasted or food-restricted rats (Ribas et al. 2012). Whilst the above studies suggest that central α2-adrenoceptor activation may be involved in the hyperphagic activity of CBG, it should be noted that recent cardiovascular safety assays in dog did not reveal any effects on cardiovascular parameters (T. Hill, personal communication), indicating that α2-adrenoceptor agonism may not be the predominant action for CBG. Given that cannabinoids acting as CB_1_R agonists have demonstrated limited clinical utility as appetite stimulants, the possibility that CBG induces hyperphagia via indirect and/or CB_1_R-independent mechanisms warrants urgent further investigation, as this pCB may represent a valuable novel therapeutic option for such applications.

A further interesting observation from the feeding experiment is the stimulation of ambulatory activity over the 2-h test duration. These data support the predicted lack of sedative effect for the 240 mg/kg dose based on results up to 120 mg/kg in the neuromotor test battery. However, they are not wholly consistent, given that a non-significant increase in activity during the feeding experiment was observed at 120 mg/kg, which was not observed in the open field test. This is not inherently contradictory however, as it is plausible that differences in test environment, and the considerably longer test duration and drug exposure time (180 vs 65 min from drug administration), allow the detection of effects too subtle to be observed in the open field.

The coincident increases in total food intake and ambulatory activity suggest the following two possible alternative interpretations of these data: that increased locomotor activity is an artefact of increased food seeking; or that increased food intake is secondary to increased activity or general arousal. For the first interpretation to be valid, any compound which increases food intake by a similar magnitude in this system would have to also increase locomotor activity levels. However, validation studies of the feeding and activity cages, using 0.5–2-mg/kg ∆^9^-THC-containing formulations, resulted in the expected stimulation of feeding behaviours but did not increase locomotor activity (unpublished observations). Given these data, and video observations showing that the majority of animals’ activities in the cages were exploratory rather than food seeking, it is apparent that the activity data do indeed represent generalised locomotor stimulation. For this locomotor stimulation to be the primary driver of increased food intake, via a general arousal mechanism, patterns of activity and food intake would have to closely mirror one another, both in terms of temporal profile and dose response. Upon close inspection of hourly intake and activity levels, it can be observed that whilst intake levels in hour 2 are very similar to hour 1 (and indeed 10 % higher in the 240-mg/kg group), activity levels in hour 2 are approximately half that in hour 1(data not shown). Further evidence of the disconnect between activity and intake can been seen in the dose response, with the highest intakes during hour 1 in the 120 mg/kg group, in contrast to the highest activity levels being in the 240 mg/kg group. These data thus argue against the interpretation that the hyperphagic activity of CBG is driven by generalised arousal, but rather that this compound directly stimulates motivation to feed, with coincident feeding-independent locomotor activation apparent at the highest dose. Whilst beyond the scope of the present study, this apparent stimulant effect of higher CBG doses warrants further investigation in models which can assess locomotor activation over extended time periods, without any confounding effects of feeding stimulation.

The tests comprising the neuromotor tolerability battery have been previously utilised for the assessment of pCBs and other drugs with known clinical neuromotor side effects. A number of drugs with known sedative effects in humans, e.g. ∆^9^-THC and benzodiazepines, elicit a sedative effect on locomotor activity in the open field test (Navarro et al. 1993; Prut and Belzung 2003). The static beam and grip strength tests have similar predictive validity for clinically observed detrimental motor effects, with both the anti-epileptic drug valproate and ∆^9^-THC-containing cannabis extracts resulting in impaired performance in these tests (Hill et al. 2012b, 2013). In contrast to ∆^9^-THC, but like the non-psychoactive pCBs CBD and cannabidiolic acid (Long et al. 2010; Brierley et al. 2016), CBG in the present study had no effect at any dose on locomotor activity in the open field test. In the static beam and grip strength tests, CBG did not elicit any detrimental effect on balance or fine motor control measures, nor on muscular strength, again in contrast to previous reports of the effects of ∆^9^-THC-containing cannabis extracts in these tests (Hill et al. 2013). To the best of our knowledge, the only published investigation of the side effect profile of CBG has been in the mouse tetrad test for classic cannabimimetic activity, in which it did not induce typical CB_1_R-mediated effects of hypomotility, catalepsy, hypothermia or analgesia up to the maximal tested dose of 80 mg/kg (El-Alfy et al. 2010). The present study thus confirms that acute CBG administration does not elicit sedation and further demonstrates that it does not have detrimental effects on balance, motor control or muscular strength.

## Conclusion

The present study demonstrates for the first time that the non-psychoactive plant cannabinoid CBG is able to stimulate appetite in pre-satiated rats, and does so without detrimental neuromotor side effects. Analysis of meal pattern microstructure revealed that CBG predominantly stimulated feeding behaviour by decreasing the latency to feed and increasing the frequency of feeding rather than by increasing individual meal size or duration. This observed meal pattern is distinct from that elicited by the major psychoactive plant cannabinoid ∆^9^-THC, suggesting that CBG acts via an as yet undetermined mechanism unlikely to be direct activation of CB_1_R. These data thus provide a rationale for pre-clinical investigation of CBG as a novel treatment in models of cancer- or chemotherapy-induced cachexia, for which there is an urgent unmet clinical need for well-tolerated appetite stimulants.

## Abbreviations

CBG: Cannabigerol
CBD: Cannabidiol
pCB: Phytocannabinoid
eCB: Endocannabinoid
∆^9^-THC: ∆^9^-tetrahydrocannabinol
CB_1_R: Cannabinoid receptor type 1
CB_2_R: Cannabinoid receptor type 2
AEA: Arachidonoylethanolamide
2-AG: 2-Arachidonoylglycerol
5-HT_1A_R: 5-Hydroxytryptamine receptor type 1A
